# Associations between Integument Color and Physical and Physiological Quality in *Pterodon pubescens* Seeds

**DOI:** 10.3390/plants11101302

**Published:** 2022-05-13

**Authors:** Renato Vieira Medeiros, Juliana de Fátima Sales, Kelly Juliane Telles Nascimento, Aurélio Rúbio Neto, Jacson Zuchi, Osvaldo Resende, Douglas Almeida Rodrigues, Arthur Almeida Rodrigues

**Affiliations:** Laboratory of Seeds, Goiano Federal Institute of Education, Science and Technology (IFGoiano), Campus Rio Verde, P.O. Box 66, Rio Verde 75901-970, Brazil; renato_biologo@hotmail.com (R.V.M.); juliana.sales@ifgoiano.edu.br (J.d.F.S.); kellytelles@gmail.com (K.J.T.N.); aurelionetorv@gmail.com (A.R.N.); jacson.zuchi@ifgoiano.edu.br (J.Z.); osvaldo.resende@ifgoiano.edu.br (O.R.); douglasalmeida_rv13@hotmail.com (D.A.R.)

**Keywords:** biochemistry, colorimetry, germination, sucupira branca, X-ray

## Abstract

*Pterodon pubescens* is a native Brazilian species typical of the cerrado biome, belonging to the Leguminosae-Papilionoideae (Fabaceae) family and popularly known as sucupira branca or faveiro. Its seeds exhibit different integument colors, which may exhibit a direct association to physiological quality related to higher germination percentage rates, vigor, and germination speed index (GSI). Therefore, selection and evaluation methods concerning *P. pubescens* seed quality are required for the preservation of this species. In this context, the aim of the present study was to determine the relationship between *P. pubescens* seed integument color and seed quality, through a combination of radiographic imaging and physiological, histochemical and biochemical assays. *P. pubescens* seeds were obtained from five matrices, and visually classified into four color classes, yellow, light brown, dark brown and black. The coordinates “L”, “a” and “b”, indicated by the colorimeter and the calculations of the chroma and hue angle values, verified that there was a difference in the color of the seeds, eliminating the subjectivity of the visual classification. Thus, the clearer the integuments, the greater their density, filling and α-amylase and β-amylase hydrolytic enzyme activities, the latter comprising important germination power indicators, in addition to high ascorbate peroxidase (APX) enzyme activities, responsible for hydrogen peroxide (H_2_O_2_) elimination. Because of this, lighter colored seeds led to higher vigor, germination and GSI rates. The removal of darker seeds from *P. pubescens* seed lots guarantees higher germination rates and vigor of new plants in nurseries destined to recompose *P. pubescens* populations.

## 1. Introduction

*Pterodon pubescens* Benth, popularly known as white sucupira or faveiro, is a native Brazilian aromatic tree, belonging to the Leguminosae-Papilionoideae (Fabaceae) family that can grow to over ten meters in height, distributed throughout the Brazilian states of Tocantins (North region), Bahia, Maranhão and Piauí (Northeast region), Mato Grosso, Mato Grosso do Sul, Goiás and the Federal District (Midwest region) and Minas Gerais and São Paulo (Southeast region), in the Amazon, Caatinga, Pantanal and Cerrado morphoclimatic domains [1,2].

*P. pubescens* exhibits high medicinal potential, as its oil nanoemulsions comprise a therapeutic resource for the treatment of several diseases, such as rheumatism, pharyngitis and respiratory inflammation and is also employed as an analgesic, depurative and tonic [3,4,5,6]. A recent study also indicated the effectiveness of its oil nanoemulsions in the topical treatment of tegumentary leishmaniasis [7].

Despite the economic potential of native species worldwide, the genetic resources of most forests are currently under serious threats due to human activities, including land use changes, forest fragmentation, the introduction of invasive species and air pollution [8,9]. In the Brazilian cerrado biome, high fire frequencies and wood exploitation for civil construction and charcoal production have increasingly reduced the richness of woody species [10]. Therefore, quality seed selection methods for subsequent seedling production are paramount for the development of high standard seed lots to enable *P. pubescens* population reforestation and recomposition efforts.

*P. pubescens* seeds exhibit a heterochrome color, ranging from light to dark. Changes in the seed integument color patterns may indicate seed quality differences [11]. Physiological quality in seeds is related to higher percentages of germination, vigor, and germination speed index (GSI). Because of this, non-destructive seed assessment methodologies, such as X-ray imaging, have been increasingly applied in the investigation of internal physical seed characteristics, seeking to quickly and effectively select higher quality seeds [12,13]. X-ray imaging combined with physiological, histochemical and biochemical assays may, therefore, guarantee the effective selection of *P. pubescens* seeds and aid in performing associations between seed color and quality.

The hydrolytic enzymes α-amylase and β-amylase act by degrading starch, which increases the levels of soluble sugars, which are primary sources of energy, maintaining cellular activities, and preventing cell death [14,15]. As they also act as reactive oxygen species (ROS) scavengers, the lack of carbohydrates can cause the formation of ROS, which are eliminated by the detoxification enzymes superoxide dismutase (SOD) and catalase (CAT) [15]. 

The biosynthesis of phenolic compounds is induced by stress and associated with the action of the enzyme phenylalanine ammonia-lyase (PAL) through the biosynthetic pathway of phenylpropanoids [15]. The accumulation of phenolic compounds favors the activity of polyphenol oxidase (PPO), which catalyzes the oxidation of phenols to quinones, which in turn produce brown quinones, a fact that can influence the color of the seed coat [16].

Although some studies are available on the potential of X-ray imaging in seed quality evaluations, most assessments have been conducted without directly linking physiological seed quality and native cerrado species preservation. To fill this knowledge gap, this study reports the first X-ray imaging data for *P. pubescens* seeds linked to their physiological quality, anatomy and biochemistry characteristics, according to integument coloration. The aim of this study was to, therefore, determine the relationship between *P. pubescens* seed color and quality, by combining radiographic images and physiological, histochemical and biochemical assays.

## 2. Results

### 2.1. Imbibition Curve

**Figure 1:**
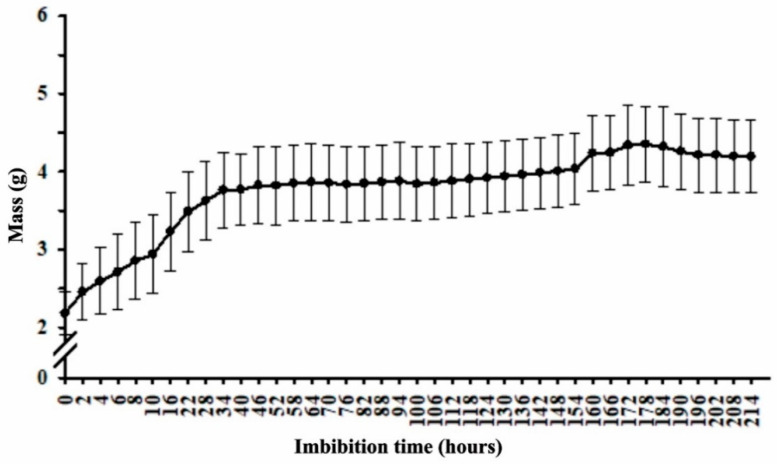
*Pterodon pubescens* seed mass gain as a function of imbibition time.

The imbibition curve of *P. pubescens* seeds reveals that water absorption followed the three-phase germination process pattern, with a constant and significant increase in seed mass during the first 40 h, comprising phase I of the germination process (Figure 1).

After 40 h, imbibition decreased and stabilized, indicating phase II (stationary) of the germination process, lasting about 150 h. Subsequently, root protrusion began, along with further water absorption, leading to a mass increase (Figure 1), characterizing phase III of the germination process.

### 2.2. Colorimetric Assessments

The results displayed in Table 1 indicate significant differences for all the analyzed variables. All the classes of subjectively classified *P. pubescens* seed colors differed regarding the “L” coordinate, which ranges from white (100) to black (zero). According to the “L” coordinate, The seeds classified as yellow exhibited the highest means, displaying a lighter hue, and the seeds classified as black exhibited the lowest means, tending to black, corroborating with the visual classification of integument applied previously. 

The coordinate “a” values were positive, indicating that *P. pubescens* seeds tend towards red. Yellow, light brown and dark brown seed classes did not differ regarding coordinate “a”, while the black seed class did, displaying a lower means. This indicates that yellow, light brown and dark browns tend more towards red than black seeds.

The coordinate “b” values were also positive, demonstrating that *P. pubescens* seeds tend towards yellow. The yellow and light brown seed classes exhibited the highest “b” means, closer to yellow than the other two color classes. In addition, the black seed class presented the lowest means, with a low trend towards yellow (Table 1).

The chroma (Cr) parameter, which comprises the relationship between “a” and “b”, was used to obtain the real color of the analyzed material and the hue angle (°h), formed between the coordinates “a” and “b”, indicating color saturation. These were not significantly different between the yellow and light brown seed classes, which exhibited the highest means, differing from the other seed color classes, with the black seed class again displaying the lowest means (Table 1).

### 2.3. Germination Test

The yellow and light brown seed classes exhibited the highest average germination percentage five days after sowing (5 DAS), compared to the dark brown and black seed classes. The yellow seeds germinated 37 and 44% and light brown seeds, 23 and 30%, more than the dark brown seeds and black seeds, respectively (Table 2).

At thirteen days after sowing (13 DAS), the final test period, the yellow and light brown seed classes again displayed superior physiological quality, differing regarding germination percentages, normal seedlings and germination speed index (GSI), compared to the dark brown and black seed classes. The germinations were 53, 48 and 60.39% higher at 13 DAS and for normal and GSI seedlings, respectively, compared to dark brown seeds, and 86, 63 and 98.89% higher for the same variables in the comparison to black seeds. Similarly, the light brown seeds exhibited 43, 36 and 58.19% higher germination rates at 13 DAS and for normal and GSI seedlings, respectively, than dark brown seeds, and 76, 51 and 98.83% higher for the same variables compared to the black seeds (Table 2).

Due to lower germination percentages, the dark brown seeds exhibited lower values regarding normal and abnormal seedlings, evidencing a lower physiological quality compared to yellow and light brown seeds. Only one black seed germinated, resulting in a normal seedling, indicating that 99% of the black seeds were killed (Table 2). The black seed that germinated did so probably because the embryo was unaffected by cotyledonary tissue deterioration, and even though a normal seedling emerged, its development would probably be inferior to lighter seeds in nature, as black seeds do not contain enough reserves for adequate plant nutrition during the early life cycle stages. Therefore, the data presented in (Table 2) indicate that the color of the *P. pubescens* tegument is directly related to the physiological quality of the seeds.

### 2.4. Radiographic Imaging

**Figure 2:**
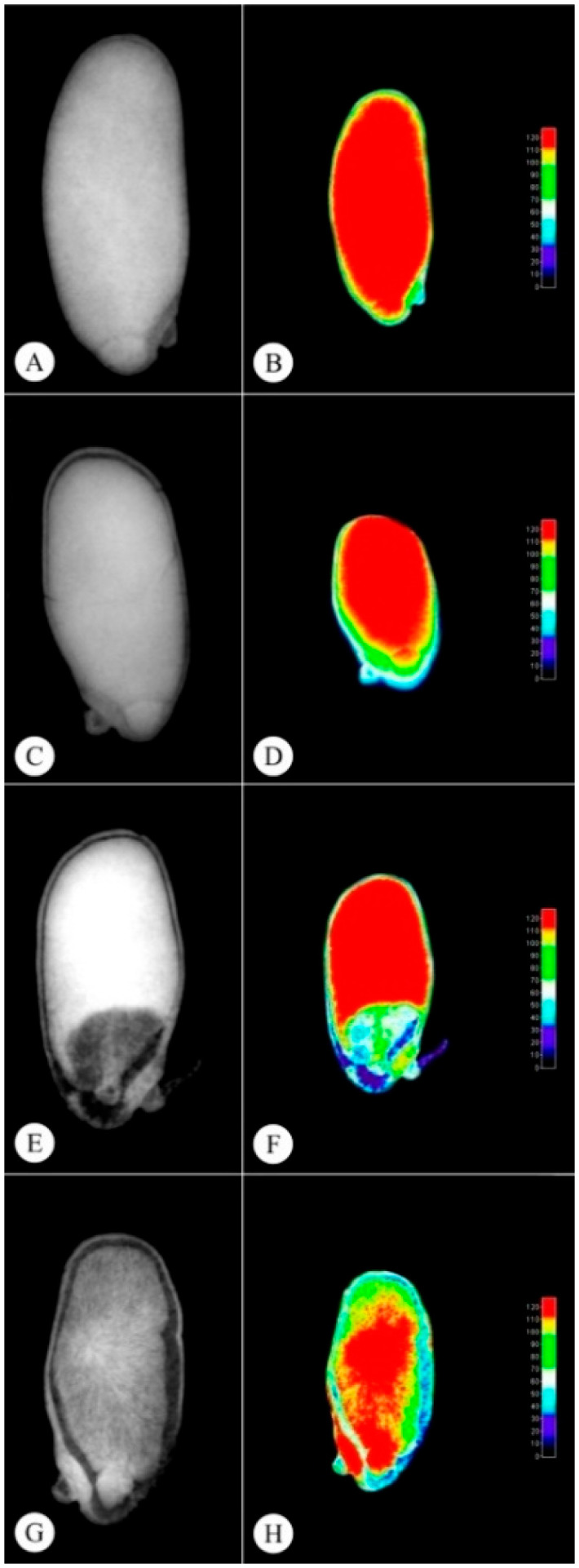
*Pterodon pubescens* radiographic seed images and 3D (**A**,**B**) yellow seeds, (**C**,**D**) light brown seeds, (**E**,**F**) dark brown seeds and (**G**,**H**) black seeds.

Original radiographic images are presented on the left of Figure 2, and tissue density histograms in gray per colored pixel in 3D are presented on the right. Warm colors are associated with high density regions and cool colors, with low density regions.

**Figure 3:**
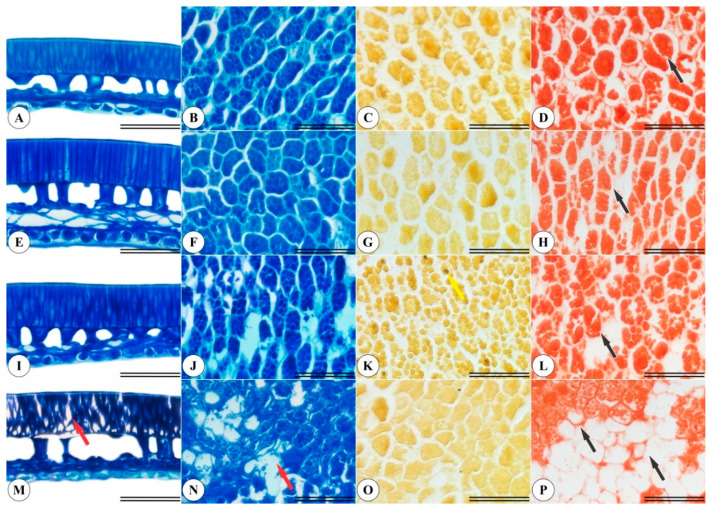
*Pterodon pubescens* seed integument and endosperm characterizations at different development stages. (**A**–**D**) yellow seeds. (**E**–**H**) light brown seeds. (**I**–**L**) dark brown seeds. (**M**–**P**) black seeds. Scale bar: 50 µm. The first column displays integument anatomy, with red arrows signaling cellular alterations. The second column displays endosperm anatomy. The yellow arrows in the third column indicate starch accumulation, and the black arrows in the fourth column indicate protein accumulation.

Yellow (Figure 2A,B) and light brown (Figure 2C,D) seeds exhibit higher physiological quality, with high ash rates, indicating higher tissue density and seed filling, resulting in higher germination rates (Table 2). The technique of image analysis of radiographic seeds is an important tool in the selection of lots with higher quality, being directly related in this study with the physiological quality (Table 2), anatomy (Figure 3), and biochemical of the seeds.

The black seeds (Figure 2G,H) exhibited lower physiological quality, with low ash rates, indicating lower seed density and filling (Table 3), resulting in 99% of non-germinated seeds (Table 2), unviable according to Figure 2G,H. On the other hand, the dark brown seeds (Figure 2E,F) exhibited higher gray levels than black seeds, resulting in 33% more germinated seeds (Table 2).

The X-ray images of the different *P. pubescens* seed color classes following ImageJ^®^ software processing and analysis were not significantly different concerning area and perimeter. The relative density, gray levels and filling data were not different among the color classes, except for the black seeds.

### 2.5. Anatomical and Histochemical Seed Characterizations 

The yellow and light brown *P. pubescens* seeds displayed differences in integument cells (Figure 3M,N) and in the endosperm region (Figure 3A,B,E,F). A small amount of starch was observed in the endosperm of the dark brown seeds (Figure 3K), marked in black by lugol. Furthermore, the yellow and light brown seeds contained higher protein levels (Figure 3D,H), while dark brown and black seeds exhibited protein extravasation in endosperm cells (Figure 3L,P), evidencing higher seed deterioration.

### 2.6. Biochemical Analyses

The light brown seeds exhibited the highest α-amylase activity means and, alongside the yellow seeds class, the highest β-amylase enzyme activity means (Table 4). These enzymes are highly associated with seed quality. Thus, the lowest α-amylase and β-amylase activity means were observed in black seeds (Table 4) and are directly associated to low physiological seed quality.

The black seed class exhibited the highest superoxide dismutase (SOD) activity means, significantly different from the other seed classes (Table 4). This implies that black seeds may be under stress, due to the toxic effects of reactive oxygen species (ROS), as SOD is an antioxidant enzyme associated with the elimination of ROS-promoted toxicity.

As for SOD, catalase (CAT) activity was the highest in the black seeds (Table 4), as SOD activity generates hydrogen peroxide (H_2_O_2_) as a by-product, followed by elimination via the CAT route, converting H_2_O_2_ in water molecules. However, the yellow and light brown seeds exhibited the highest ascorbate peroxidase (APX) activity means, differing from the dark brown seeds and undetected in the black seeds. APX is also responsible for H_2_O_2_ elimination, with higher activity means detected in the yellow and light brown seeds, which may be associated to the storage of these enzymes in cellular compartments to eliminate H_2_O_2_ when under stress, which would explain lower APX detection in dark brown seeds and non-detection in black seeds.

Peroxidase (POX) activity was higher in black seeds, significantly different from the other seed classes (Table 5). This suggests that POX is more active in black seeds, catalyzing the oxidation of substrates, such as phenolic compounds and the amino acid tyrosine, using the oxidizing power of H_2_O_2_. This activity may be associated to the darkening of *P. pubescens* seeds during deterioration.

The yellow seeds exhibited the highest polyphenoloxidase (PPO) activity means, significantly differing from other seed color classes (Table 5). PPO is stored in vacuoles and catalyzes enzymatic browning reactions during the deterioration process. Thus, its lower detection in the light brown, dark brown and black seed classes indicates that PPO activity had already occurred, oxidizing phenolic compounds and producing quinones, which, in turn, react with proteins and with PPO itself, contributing to seed darkening.

The highest amounts of proteins were detected in the yellow, light brown and dark brown seed classes (Table 5), agreeing with Fig. 3p, which revealed protein-free cells in the black seeds.

Malondialdehyde (MDA) content in black seeds was higher compared to the other seed classes (Table 5), probably due to lipid peroxidation. Thus, black seeds are continuously in oxidative stress conditions.

**Figure 4:**
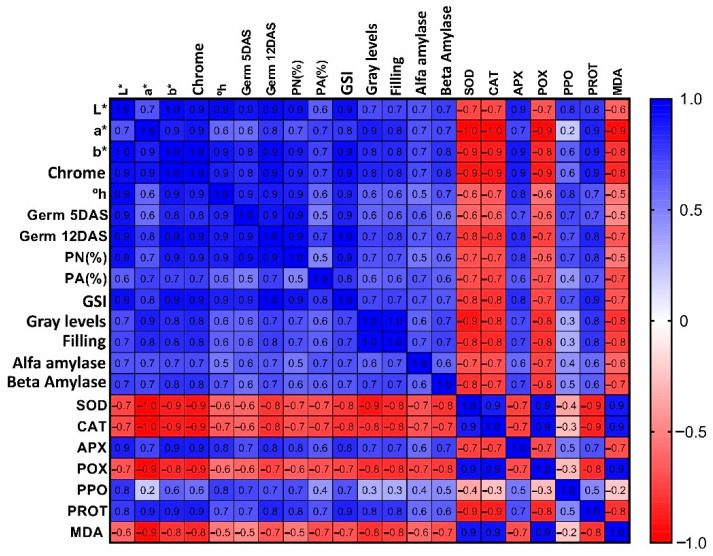
Heatmap plot of Pearson’s correlation for all the significant variables. L* (luminosity), a* (color index “a”), b* (color index “b”), Chrome, °h (hue color angle), germ 5 DAS (germination percentage at five days after sowing), germ 12 DAS (germination percentage at thirteen days after sowing), PN (%) (percentage of normal seedlings), PA (%) (percentage of abnormal seedlings), GSI (germination speed index), gray levels, filling, alfa amylase, beta amylase, SOD (superoxide dismutase), CAT (catalase), APX (ascorbate peroxidase), POX (peroxidase), PPO (polyphenol oxidase), PROT (protein) and MDA (malondialdehyde).

The heatmap graph exhibited in Figure 4 indicates that, the higher the “L”, “a” and “b” values, the higher the germination rates at 5 and 12 DAS, GSI, normal seedlings, gray levels, filling, α-amylase, β-amylase, APX and PPO, and total proteins. These parameters are inversely proportional to SOD, CAT, POX and MDA (Figure 4). Thus, the black seeds exhibit higher enzymatic activity associated to stress and compound deterioration.

## 3. Discussion

The seed germination process consists of embryonic axis growth commencement. This process can be assessed by analyzing seed imbibition curves, which usually present a three-phase pattern. The first phase comprises rapid seed imbibition, the second (stationary) phase, intense hydration and breathing intensity reductions, and the third, stabilization. The end of an imbibition curve begins with the disruption of the seed coat by the embryo’s radicle, when a new and slight water absorption occurs due to the metabolic requirements of embryo cell divisions and elongations, characterizing the third phase of the germination process [17,18,19].

A seed can present integument color variations, associated to maturation degree and/or tissue oxidation [20]. Therefore, the difference in the hues observed in the seeds of a certain species may indicate physiological quality differences [21]. The seeds of *P. pubescens* probably have color variations in the integument due to different degrees of maturation, as the fruits are indehiscent and it was observed that they can remain connected to the mother plant for a long period of time after fruiting; therefore, the seeds of the first fruits age and deteriorate first, in relation to the fruits produced at the end of the fruiting period.

The color difference between seeds can be identified by a ColorFlex EZ colorimeter employing the Hunter color system, where L (luminosity) values near 100 tend to white and, close to 0, to black. For “a”, positive values tend to red and negative values to green, and for “b”, positive values tend to yellow and negative to blue. Thus, “L”, “a” and “b” allow for chroma and hue calculations, which identify the real color of the investigated object and its degree of saturation, respectively [22]. 

Lighter seeds from the same angiosperm species generally display better physiological quality, leading to better germination and GSI percentages. Thus, seed color is an important seed quality indicator, resulting in the production of more vigorous seedlings [23,24]. The same was observed in this study, where the seeds with lighter tegument presented the best physiological quality indices, confirmed by the germination test and GSI values (Table 2).

The radiographic seed images, processed and analyzed by the ImageJ^®^ software, aided in the identification of seeds containing healthy embryos and adequate filling, according to tissue density in gray per pixel, with higher gray levels indicating higher physiological viability and vigor [13]. The methodology of radiographic images was used in a fast and non-destructive way to classify the seeds of *P. pubescens*, providing important information about the physical and physiological parameters and agreeing with the author of the study of [25], who used X-ray images to rate the quality of *Crambe abyssinica* seeds. 

*Pterodon emarginatus* Vogel, considered a sister species to *P. pubescens*, presents expressive endosperm protein and lipid contents and rare starch grains [26], similar to the findings reported herein for starch (Figure 3K) and protein content (Figure 3D,H,L,P). The present study also detected integument cell disarrangement in the seeds that exhibited greater deterioration (Figure 3M), as well as endoplasmic content extravasation (Figure 3N) and lower protein accumulation (Figure 3P).

Hydrolase enzymes are associated with protein body degradation during the germination or degradation phase in orthodox seeds [27,28]. The seeds of some dead endosperm cereal species exhibit a live cell layer (aleurone layer), which produces digestive enzymes responsible for endosperm reserve mobilization [17].

Gibberellin production by plant embryos during the germination process promote the development of hydrolytic enzymes α-amylase and β-amylase, which are responsible for the degradation of carbohydrates found in seed reserve tissues for use by the developing embryo, resulting in greater accumulation of these enzymes in healthy seeds [14].

Carbohydrates (starch and soluble sugars) are primary energy reserve sources. The increase in the concentration of soluble sugars in the seeds is due to the action of the hydrolytic enzymes α-amylase and β-amylase that break down the starch, which can promote respiratory activity, improving the levels of adenosine triphosphate (ATP), which in turn facilitate process maintenance, delaying cell death [14,15]. Therefore, the classes of yellow seeds and light chestnuts are healthier because they have a higher concentration of these enzymes (Table 4). 

In addition, soluble carbohydrates can act as scavengers of reactive oxygen species (ROS), preserving the integrity of the membranes. On the other hand, the lack of carbohydrates can cause the formation of ROS. A higher concentration of ROS raises the levels of detoxification enzymes superoxide dismutase (SOD) and catalase (CAT), which are responsible for eliminating ROS [15]. The possibility of a higher concentration of ROS explains the high levels of SOD and CAT in the black class seeds (Table 4).

SOD is a metalloenzyme that belongs to the intracellular antioxidant enzymatic system and its activity is associated with stress tolerance in plants, as the first line of defense against the toxic effects of high levels of ROS [29,30]. SOD activity produces hydrogen peroxide (H_2_O_2_) through superoxide dismutation, which is then converted into water molecules by CAT, located in glyoxisomes and peroxisomes [31]. In turn, scorbate peroxidase (APX) and other enzymes that take part in the glutathione-ascorbate cycle are present in chloroplasts, cytoplasm, mitochondria, peroxisomes and apoplasts, and are also capable of eliminating H_2_O_2_ through the glutathione-ascorbate cycle [32]. APX activity in seeds may be associated with the ability of a certain species to develop ways to tolerate stress, mainly eliminating ROS [33,34].

According to Freitas [35], POX can catalyze many oxidative reactions in plants using H_2_O_2_ as a substrate, or O_2_ as a hydrogen acceptor. This enzyme is also associated with plant tissue darkening [36], which may have been the case for *P. pubescens* seeds investigated herein.

Damage to the seed membrane system can trigger enzymatic mechanisms, altering cell constituents and contributing to the contact of polyphenol oxidase (PPO), which occurs exclusively in plastids with phenolic compounds that are stored in vacuoles, making phenol oxidation inevitable, which in turn, when converted to quinones, may react with proteins and with PPO itself [37], explaining the higher amount of total proteins in the lighter *P. pubescens* seeds detected herein (Table 5).

Malondialdehyde (MDA), the end product of lipid peroxidation, is higher in deteriorating seeds. Consequently, seeds under physiological stress exhibit higher MDA concentrations [38], which explains the greater accumulation of MDA in black seeds observed herein.

## 4. Material and methods

### 4.1. Sampling

*P. pubescens* fruits were sampled directly from the branches of five matrices located in the cerrado biome, in the municipality of Montes Claros de Goiás, Brazil, in March 2019. After sampling, the selected seeds were separated from the *P. pubescens* fruits, with the aid of cutters at the Federal Goiano Institute Seed Laboratory, Rio Verde campus. The initial seed water content was determined by the oven method, maintaining the seeds at 105 ± 3 °C for 24 h, according to Brazil [39]. Two five-seed replicates, corresponding to approximately three grams, with a water content of 7.65% on a wet basis were assessed. The different integument color patterns, possibly associated to seed maturation degree, were noted. The seeds were then visually separated into four color classes, as yellow, light brown, dark brown and black.

### 4.2. Imbibition Curve Test

In order to understand the imbibition behavior of *P. pubescens* seeds, the imbibition curve test was carried out with the yellow seeds, as the other color classes did not constitute enough seeds for all the tests carried out in this study. Four twenty-five-seed repetitions were placed on two germitest sheets moistened with distilled water at 2.5 times the mass of the sheets inside germination boxes and weighed at two-hour intervals during the first twenty-four hours and every six hours until the end of the imbibition test. The weightings were completed 214 h after the beginning of the test, when 50% of the seeds presented root radicle protrusions of at least 1 mm. The results were expressed as mass gain in graph form.

### 4.3. Colorimetric Assessments

A colorimetric test was carried out, employing a completely randomized design through the direct assessment of the reflectance values of the “L”, “a” and “b” coordinates, employing a ColorFlex EZ colorimeter (Hunter Associates Laboratory Inc., Sunset Hills Road, Reston, VA) using the Hunter color system. “L” indicates white and black; “a”, red and green; and “b”, yellow and blue. One hundred seeds from each color class were analyzed (8.49 g for yellow seeds, 7.66 g for light brown seeds, 6.30 g for dark brown seeds and 3.62 g for black seeds). The seed mass was homogenized after the first reading was taken for each color class and a new reading was performed, totaling five homogenization and color determination cycles.

Subsequently, chroma (Cr) and hue color angle (°h) values were calculated, according to Equations (1) and (2), respectively, eliminating the subjectivity of visual assessments [40].

(1) $$\mathrm{Cr}=\left[{\left(a^{2}+b^{2}\right)}^{0.5}\right]$$

(2) $${}^{\circ}h=\left[\mathrm{arctang}\left(\frac{b}{a}\right)\right]$$

where Cr comprises the chroma; °h, is the hue angle; and L, a and b are the coordinates determined by the colorimeter.

### 4.4. Radiographic Images

The X-ray test was performed applying a completely randomized design comprising five replications, usuing an LX-60 device (Faxitron X-Ray Corporation, model 43855A, Hologic, Britannia Drive, Tucson, Arizona USA). *P. pubescens* seeds presenting different colors were previously fixed on transparent paper with double-sided tape and sequentially numbered, totaling 524 yellow seeds, 133 light brown seeds, 100 dark brown seeds and 244 black seeds and radiographed at 27 Kvs for 10 s. The saved images were analyzed using the ImageJ^®^ software version 1.8 to obtain area, perimeter, relative density, median gray and filling data. 

### 4.5. Physiological Assays

As a pre-germinative treatment, the seed integuments of the opposite side of the embryos were trimmed with the aid of a scalpel, as *P. pubescens* seeds exhibit integumentary dormancy [41]. Seed germination was performed on germitest paper sheet rolls previously cut to 20 cm × 15 cm and autoclaved, each accommodating only one seed. This permits radiographic image comparisons, as each seed numbered for the X-ray test carried the same numbering as the individualized germitest paper sheets rolls. 

The germitest paper sheets were moistened with distilled water at 2.5 times the sheet mass [30]. Each seed was placed on two moistened germitest sheets and covered with a third sheet to prepare the paper rolls. The germination test was carried out in a completely randomized design, comprising five replications. Each repetition consisted of a plastic bag containing twenty rolls placed in a germination chamber at 25 °C, over a 12-h photoperiod. The adopted botanical criterion considered germinated seeds as the seeds that displayed 1.0 mm radicles. The treatments were evaluated on the 5th day after sowing (DAS) for both the germinated seeds and non-germinated seeds and on the 13th DAS for germinated seeds, non-germinated seeds, normal seedlings and abnormal seedlings.

The germination speed index (GSI) was obtained by calculating the sum of seeds germinated each day divided by the number of days elapsed between sowing and germination, according to Maguire [42].

### 4.6. Morphoanatomical Seed Characterization

For the morphoanatomical analyses, 2 cm^2^ samples were obtained from the endosperm region of *P. pubescens* seeds from each color class subsequently fixed in Karnovsky’s solution [43] for 24 h. The samples were then pre-washed in a phosphate buffer (0.1 M, pH 7.2) and dehydrated in an increasing ethanol series (30% to 100%) and, finally, pre-infiltrated and infiltrated in Historesin (Leica, Wetzlar, Germany), as per the manufacturer’s recommendations. The samples were then sectioned transversely using a rotating microtome (Model 1508R, Logen Scientific, Shanghai, China), obtaining 5 μm thick sections and stained with toluidine blue-polychromatic staining (0.05% 0.1 M phosphate buffer, pH 6, 8) [44]. Starch was detected by histochemical staining with a 10 g L^−1^ lugol solution [45]. Total protein detection was performed through Xylidine ponceau (XP) staining [46]. Images were obtained using an Olympus microscope (BX61, Tokyo, Japan), coupled to a DP-72 camera, using the brightfield option.

### 4.7. Determination of Antioxidant System Enzymatic Activity

Samples containing two *P. pubescens* seeds from each color class were stored individually in aluminum foil, emerged in liquid nitrogen (N_2_) for instantaneous freezing and stored in an ultrafreezer at −80 °C until further analysis.

The enzymatic extract used to determine α-amylase (EC 3.2.1.1) and β-amylase (EC 3.2.1.2), superoxide dismutase (SOD) (EC 1.15.1.1), catalase (CAT) (EC 1.11.1.6), ascorbate peroxidase (APX) (EC 1.11.1.11), peroxidases (POX) (EC 1.11.1.7) and polyphenoloxidase (PPO) (EC 1.10.3.1) activities was obtained by crushing 0.250 g of seeds with liquid N_2_ and homogenization in 2 mL of a 50 mM potassium phosphate buffer (pH 6.8), containing 0.1 mM ethylenediaminetetraacetic acid (EDTA), 1 mM phenylmethylsulfonyl fluoride (PMSF) and 5% (m/v) polyvinylpyrrolidone (PVPP). The homogenates were maintained overnight for 14 h at 10 °C and then centrifuged at 12,000× *g* for 15 min at 4 °C. The final supernatants were then used as the extract for enzymatic determinations. A completely randomized experimental design, comprising five replications for each enzyme, was applied.

α-amylase activity was determined by adding 250 μL of the enzymatic extract to a reaction medium containing 150 μL of 3 mM CaCl_2_ and incubated at 70 °C for 5 min to inactivate β-amylase. The 250 μL aliquot of β-amylase inactivated extract was then mixed with 500 μL of 100 mM sodium citrate buffer, pH 5.0 and 250 μL of 1% starch solution and incubated at 30 °C. The reaction was stopped after 5 min, by adding 2 mL of the reading reagent (3,5-dinitrosalicylic acid 1% (DNS), 2M NaOH and potassium sodium tartrate) and heating the extract at 50 °C for 5 min, followed by dilution with 4 mL of distilled water [47,48,49].

β-amylase activity was determined by adding 180 μL of the enzymatic extract to a reaction medium containing 110 μL of 0.1 M EDTA to inactivate α-amylase. The 250 μL aliquot of α-amylase inactivated extract was mixed with 500 μL of 100 mM sodium citrate buffer, pH 5.0 and 250 μL of a 1% starch solution and incubated at 30 °C for 5 min. Subsequently, 2 mL of the reading reaction (3,5-dinitrosalicylic acid 1% (DNS), 2M NaOH and potassium sodium tartrate) were added, followed by heating at 50 °C for 5 min and the same dilution as described above.

The reducing sugars formed by α- and β-amylase action were quantified at 540 nm and content calculations were performed using a 0.5 mg/mL standard maltose curve [47,48,49].

SOD activity was determined by adding 60 μL of the enzymatic extract to a reaction mixture containing 50 mM sodium phosphate buffer (pH 7.8), 13 mM methionine, p-nitro-tetrazolium blue (NBT) 75 µM, 0.1 mM EDTA and 2 µM riboflavin [50], totaling 2 mL. The reaction took place at 25 °C under 15 W lamps. After 10 min of light exposure, the lights were turned off and the blue formazan, produced by NBT photoreduction, was determined at 560 nm using a spectrophotometer (Evolution 60, Thermo Fisher Scientific Inc., Waltham, Massachusetts, USA) [51]. One unit of SOD was defined as the amount of enzyme required to inhibit NBT photoreduction by 50% [52]. SOD activity was expressed as µmol min^−1^ mg^−1^ of protein.

Catalase activity (CAT) was determined according to Cakmak and Marschner [53], applying a molar extinction coefficient of 36 M^−1^ cm^−1^ and expressed as µmol min^−1^ mg^−1^ of protein.

APX activity was determined according to Nakano and Asada [54]. The reaction mixture comprised 50 mM potassium phosphate buffer (pH 6.8), 1 mM H_2_O_2_ and 0.8 mM ascorbate in 1 mL. The reaction began by adding 15 μL of the enzymatic extract to the reaction mixture and determining the ascorbate-dependent oxidation of H_2_O_2_ at 290 nm for 1 min at 25 °C. APX activity was determined using a molar extinction coefficient of 2.8 mM^−1^ cm^−1^ and expressed as µmol min^−1^ mg^−1^ of protein.

POX activity was determined by pyrogallol oxidation according to Kar and Mishra [55]. The reaction mixture contained 25 mM potassium phosphate buffer (pH 6.8), 20 mM pyrogallol and 20 mM H_2_O_2_ in 2 mL. The reaction began by adding 15 µL of the enzymatic extract to the reaction mixture and determining the consumption of H_2_O_2_ at 420 nm, for 1 min at 25 °C. POX activity was determined using a molar extinction coefficient of 2.47 mM^−1^ cm^−1^ and expressed as µmol of purpurogallin produced min^−1^ mg^−1^ of protein. PFO activity was determined similarly to POX, with the exception of H_2_O_2_, which was omitted from the reaction mixture.

Polyphenol oxide (PPO) activity was determined according to Kar and Mishra [55], using a molar extinction coefficient of 2.47 mM^−1^ cm^−1^ and expressed as µmol of purpurogallin produced µmol min^−1^ mg^−1^ of protein.

The total protein concentrations in each sample were determined by adding 10 µL of the crude extracts used to determine enzymatic activities to 1190 µL of Bradford solution, followed by absorbance determinations at 595 nm [56], expressed as g g^−1^.

Cellular damage was assessed through lipid peroxidation determinations using MDA as the standard, according to Cakmak and Horst [57]. Seed samples from each repetition were ground in liquid N_2_ using a mortar until obtaining a fine powder, which was then homogenized in 2 mL of 1% trichloroacetic acid (TCA) (*w*/*v*) and centrifuged at 12,000× *g* for 15 min at 4 °C. After centrifugation, 0.5 mL of the supernatant were mixed with 1.5 mL of a 0.5% (*m*/*v*) thiobarbituric acid solution (prepared in 20% (*m*/*v*) TCA) and incubated in a water bath at 95 °C, for 30 min. The reaction was then stopped in an ice bath, followed by centrifugation at 9000× *g* for 10 min and specific absorbance determination of the supernatants at 532 nm. Nonspecific absorbances were measured at 600 nm and subtracted from the specific absorbance values. MDA concentrations were calculated using an extinction coefficient of 155 mM^−1^ cm^−1^ and expressed as µmol kg^−1^ of fresh mass [58]. 

### 4.8. Statistical Analyses

An analysis of variance using the F test was applied to the obtained data. When significant effects were observed, the means were compared using the Tukey test (*p* ≤ 0.05), employing the Sisvar^®^ statistical software [59]. Pearson’s correlation was applied to the variables that exhibited significant effects and a heatmap graph was generated using the GraphPad Prism^®^ 8.0 software (GraphPad Prism Inc., San Diego, CA, USA).

## 5. Conclusions

Integument seed color in *P. pubescens* is directly associated to physiological seed quality. Radiographic seed imaging comprises an important tool in the selection of higher quality seed lots, as the findings can be directly associated to the physiological, anatomical and biochemical seed quality. The higher activity detected for α-amylase and β-amylase in light-colored seeds is an important germination indicator, alongside high APX activity, responsible for H_2_O_2_ elimination, in contrast to the black seed class, which exhibited high SOD, CAT and POX activities and MDA content, important stress indicators.

In sum, this study revealed important findings concerning the preservation and survival of *P. pubescens*. It is important to note that the removal of dark seeds from seed *P. pubescens* lots guarantees higher germination and vigor rates of new plants, thus, increasing the production of vigorous seedlings in plant nurseries destined for the recomposition of this species.

## Figures and Tables

**Table 1:** Luminosity (L), color index “a” (a), color index “b” (b), chroma (Cr) and hue color angle (°h) in *Pterodon pubescens* seeds, subjectively categorized into different integument color classes.

Seed Class	L	a *	b *	Cr	°h
Yellow	36.19 ± 0.305 a	8.05 ± 0.125 a	22.88 ± 0.517 a	24.26 ± 0.511 a	1.53 ± 0.001 a
Light brown	32.24 ± 0.462 b	9.00 ± 0.254 a	20.08 ± 0.248 a	22.01 ± 0.218 a	1.47 ± 0.001 a
Dark brown	23.89 ± 0.435 c	8.64 ± 0.669 a	12.44 ± 1.824 b	15.18 ± 1.820 b	1.32 ± 0.040 b
Black	20.58 ± 0.181 d	1.44 ± 0.275 b	1.63 ± 0.193 c	2.17 ± 0.327 c	0.39 ± 0.041 c
**One-Way ANOVA**					
**F (*t*-test)**	395.13 *	85.19 *	97.86 *	105.80 *	315.27 *
***P***	**<0.0000**	**<0.0000**	**<0.0000**	**<0.0000**	**<0.0000**
***CV (%)***	**2.23**	**9.92**	**11.67**	**10.52**	**4.43**

Mean ± SE (*n* = 3), means followed by the same lowercase letters in the column did not differ at 5% (*) significance using the Tukey test.

**Table 2:** Germination percentage at five days after sowing (G% 5 DAS), germination percentage at thirteen days after sowing (G% 13 DAS), percentage of normal seedlings (PN%), percentage of abnormal seedlings (PA%) and GSI (germination speed index) for *Pterodon pubescens* categorized into different color classes.

Seed Class	G% 5 DAS	G% 13 DAS	PN%	PA%	GSI
Yellow	45 ± 5.701 a	87 ± 4.062 a	64 ± 6.403 a	23 ± 6.042 a	3.61 ± 0.166 a
Light brown	31 ± 4.848 a	77 ± 6.245 a	52 ± 6.042 a	25 ± 5.244 a	3.42 ± 0.394 a
Dark brown	8 ± 2.550 b	34 ± 6.782 b	16 ± 4.301 b	18 ± 4.062 ab	1.43 ± 0.253 b
Black	1 ± 1.000 b	1 ± 1.000 c	1 ± 1.000 b	0 ± 0.000 b	0.04 ± 0.040 c
**One-Way ANOVA**					
**F (*t*-test)**	26.14 *	61.85 *	36.22 *	6.44 *	46.85 *
***P***	**0.0000**	**0.0000**	**0.0000**	**0.0046**	**0.0000**
***CV (%)***	**41.93**	**22.75**	**33.12**	**60.80**	**26.20**

Mean ± SE (*n* = 5), means followed by the same lowercase letters in the column did not differ at 5% (*) significance using the Tukey test.

**Table 3:** Area, perimeter, relative density, gray levels and filling values for yellow, light brown, dark brown and black *Pterodon pubescens* seeds.

Seed Class	Area (mm^2^)	Perimeter (mm^2^)	Relative Density (Gray.Pixel^−1^)	Gray Levels (Gray.Pixel^−1^)	Filling (%)
Yellow	20.605 ± 0.4164 a	19.079 ± 0.2325 a	127.911 ± 1.4009 a	138.20 ± 1.6553 a	98.35 ± 0.0441 a
Light brown	19.713 ± 0.3781 a	18.616 ± 0.2562 a	127.049 ± 2.6518 a	138.60 ± 2.3152 a	98.10 ± 0.2027 a
Dark brown	17.642 ± 0.9103 a	17.285 ± 0.4826 a	121.843 ± 2.0572 a	132.00 ± 2.0976 a	96.20 ± 0.8651 a
Black	19.550 ± 2.0304 a	18.207 ± 1.0513 a	88.969 ± 9.0533 b	90.20 ± 9.9870 b	88.21 ± 2.2419 b
**One-Way ANOVA**					
**F (*t*-test)**	**1.18 ^ns^**	**1.60 ^ns^**	14.40 *	19.23 *	15.63 *
***P***	**0.3485**	**0.2295**	**0.0001**	**0.0000**	**0.0000**
***CV (%)***	**13.24**	**7.38**	**9.37**	**9.49**	**2.83**

Mean ± SE (*n* = 5), means followed by the same lowercase letters in the column did not differ at 5% (*) significance using the Tukey test.

**Table 4:** α-amylase (mg maltose/mg protein), β-amylase (mg maltose/mg protein), superoxide dismutase (SOD) (unit of SOD/min/mg protein) and catalase (CAT) (µmol/min/mg of protein) activities in different *Pterodon pubescens* seed classes.

Seed Class	α-Amylase	β-Amylase	SOD	CAT
Yellow	9.41 ± 1.330 ab	12.51 ± 0.284 a	1.59 ± 0.096 b	2.91 ± 0.249 b
Light brown	9.98 ± 1.633 a	10.62 ± 2.031 a	1.56 ± 0.024 b	2.78 ± 0.233 b
Dark brown	7.80 ± 0.602 ab	9.89 ± 1.116 ab	1.98 ± 0.098 b	3.31 ± 0.411 b
Black	4.79 ± 0.764 b	5.00 ± 0.727 b	7.68 ± 0.959 a	13.11 ± 1.356 a
**One-Way ANOVA**				
**F (*t*-test)**	4.03 *	6.86 *	38.16 *	48.29 *
***P***	**0.0339**	**0.0060**	**0.0000**	**0.0000**
***CV (%)***	**29.00**	**25.72**	**30.23**	**26.34**

Mean ± SE (*n* = 4), means followed by the same lowercase letters in the column did not differ at 5% (*) significance using the Tukey test.

**Table 5:** Ascorbate peroxidase (APX) (nmol/min/mg protein), peroxidase (POX) (µmol/min/mg protein), polyphenol oxidase (PPO) (nmol/min/mg protein), protein (PROT) (mg/mg of fresh mass) and malondialdehyde (MDA) (µmol/mg of fresh mass) in different *Pterodon pubescens* seed classes.

Seed Class	APX	POX	PPO	PROT	MDA
Yellow	66.05 ± 13.837 a	0.09 ± 0.017 b	9.22 ± 0.358 a	0.22 ± 0.016 a	49.46 ± 2.032 b
Light brown	70.97 ± 8.884 a	0.12 ± 0.029 b	6.29 ± 0.700 b	0.18 ± 0.029 a	50.81 ± 1.050 b
Dark brown	27.40 ± 4.630 b	0.09 ± 0.005 b	4.12 ± 0.349 b	0.17 ± 0.002 a	47.03 ± 4.024 b
Black	0.00 ± 0.000 b	0.48 ± 0.041 a	5.21 ± 0.688 b	0.06 ± 0.011 b	75.85 ± 0.652 a
**One-Way ANOVA**					
**F (*t*-test)**	15.49 *	51.04 *	15.84 *	15.15 *	33.21 *
***P***	**0.0002**	**0.0000**	**0.0002**	**0.0002**	**0.0000**
***CV (%)***	**41.56**	**27.47**	**17.74**	**22.72**	**8.38**

Mean ± SE (*n* = 4), means followed by the same lowercase letters in the column did not differ at 5% (*) significance using the Tukey test.
